# Supplementary material for: EEG p-adic quantum potential accurately identifies depression, schizophrenia and cognitive decline
Source: PLoS One. 2021 Aug 5;16(8):e0255529. doi: 10.1371/journal.pone.0255529 (PMC8341571; doi:10.1371/journal.pone.0255529)
Supplement: Attachment — Submitted filename: PONE-D-21-10465_Report.pdf [file pone.0255529.s001.pdf]

Report on "EEG p-Adic Quantum Potential Accurately Identifies Depression, Schizophrenia and Cognitive Decline by Benninger et al"

The paper introduces new a quantum potential mean and variability score (qpmvs), to identify neuropsychiatric and neurocognitive disorders with high accuracy, based on EEG recordings. It is fascinating, highly interdisciplinary, paper involving quantum physics, neurophysiology, among other areas. My report is dedicated only to the physics/mahematics of the qpmvs. In my view, the description of the underlying physical model requires serious improvements. I suggest including an appendix explaining the model and its numerical implementation.

Let me mention just a few concerns:

1. p is used to denote a p-adic number and p-value (statistics), which creates a huge confusion. From the introduction " coupled with p**-adic numbers** and quantum theory. Based on area under the curve, high accuracy was obtained in separating healthy controls from those diagnosed with schizophrenia ($p < 0.0001$)." It seems that $p = 2$!

2. The authors do not use commas to describe intervals, which creates a lot confusion. For instance, it is really difficult to read the formula

$$t \in [\,1 \;\; floor(\frac{no. of\ data\ points}{sample\ rate})/(window\ duration)]$$

3. The computation of the quantum potential requires the numerical computation of derivatives of functions having jumping-type singularities, these derivatives naturally contain Dirac delta functions. The numerical implementation of the formulae presented in the paper deserves a discussion. The authors do not provide details of the implementation. The MATLAB code is supposed be available in www.ramot.org, this is a very big website, I could not find the mentioned code.

4. It is important to provide some details about the many averages used in the paper: participant, group, etc. The authors should remember the paper is addressed to a large scientific community. I cannot understand formulas of type What is the meaning of the symbol $\vee$?

$$\text{qpmvs}_{patient} = mIqp_{patient} * \sum (log10|\int (Qpdq)_t|)_{patient} > mIqp_{group} + sIqp_{groups} \vee$$

$$(log10|\int (Qpdq)_t|)_{patient} > mIqp_{group} - sIqp_{groups}$$
