# Supplementary material for: EEG p-adic quantum potential accurately identifies depression, schizophrenia and cognitive decline
Source: PLoS One. 2021 Aug 5;16(8):e0255529. doi: 10.1371/journal.pone.0255529 (PMC8341571; doi:10.1371/journal.pone.0255529)
Supplement: Attachment — Submitted filename: Response to Reviewers.docx [file pone.0255529.s002.docx]

**Response to Reviewers** - EEG p-adic quantum potential accurately identifies depression, schizophrenia and cognitive decline

Dear Reviewers,

Thank you very much for your encouraging comments and your great help to improve the manuscript. According to your suggestions, we made appropriate changes highlighted in the manuscript with markups.

We added a section in the discussion to comply with the constructive comments from reviewer number one. The inclusion of healthy control patients was discussed as limitation complying with the very well-placed comment (“One limitation of our study is related to the heterogenic group of healthy controls. This group is comprised of patients which underwent routine EEG without a clear indication and no neuro-psychiatric disorders but might include those with unspecific headaches including migraine and tension-type headache or dizziness. Currently we cannot exclude an influence of those complaints on the qpmvs.”

The manuscript was also edited to improve language.

We would like to thank the second reviewer for his constructive remark regarding the use of the same symbol (p) for two different mathematical entities. This might have caused a disturbance by the reader. As the letter p is standard for both areas, statistics and number theory we decided to use a bold and italic fond when referring to ***p***-adic and now expect a clearer distinguishability between these two notations. As the comment on the use of 2-adic numbers throughout the paper, we added a remark (parentheses 2 last lines of page 4). We would like to preserve the presentation for an arbitrary ***p***, since it can be useful in some applications. In this context, “Q_p” for the field of p-adic numbers, was exchanged with both letters as capital “QP”.

We further re-wrote the methods part regarding the 2-adic quantum potential calculation sub-section and the section describing the quantum potential mean and variability score (qpmvs). To improve the understanding regarding the 2-adic quantum potential calculation, we added as suggested a series of algorithm steps with detailed explanations regarding each step and improved the mathematical terms in each equation. Moreover, we defined rigidly the averages and standard deviation terms. These changes have been made to meet with the remarks #2 and #4 of reviewer number 2.

We also like to comment on the seconds reviewer remark #3 (“The computation of the quantum potential requires the numerical computation of derivatives of functions having jumping-type singularities, these derivatives naturally contain Dirac delta functions”). As can be seen from the changes in the 2-adic quantum potential calculation sub-section, step 9, equations 5-7, the numerical calculation do not involve such Dirac delta functions. Moreover, the meaning of the QP function is explained in step 9 as “Thus, the ambiguous quantum potential notion becomes in our framework quite trivially a score or measure of hierarchical topology”. This meaning of the QP is a direct implication of our numerical 2-adic quantum potential computation but can be extended to the non-numerical cases.

The data availability was updated and improved and the original data files are anonymized and available as a three-dimensional matrix as a matlab file at <https://datadryad.org/stash/share/AWmC0-Afzx29cOkYDXQ6y2-7HF4GBvG-J-9i8hDQZsw> which has been added to the manuscript.
